# Epidemiological characteristics and risk factors for severity of product-related injuries in children during COVID-19: a non-pharmaceutical intervention study

**DOI:** 10.3389/fpubh.2026.1783277

**Published:** 2026-02-20

**Authors:** Jie Chen, Yanqi Lan, Zhuoping Zhang, Qionghua Zhang, Youlan Chen, Zhinan Guo, Jinhua Zhang

**Affiliations:** 1 Xiamen Center for Disease Control and Prevention, Xiamen, Fujian, China; 2 Huli District Center for Disease Control and Prevention, Xiamen, Fujian, China; 3 Jimei District Center for Disease Control and Prevention, Xiamen, Fujian, China

**Keywords:** children, COVID-19, injury severity, interaction analysis, non-pharmacological interventions, product injury

## Abstract

**Introduction:**

This study utilized the “natural experiment” created by the COVID-19 pandemic to assess the impact of non-pharmaceutical interventions (NPIs) on the epidemiological characteristics and determinants of the severity of product-related injuries among children.

**Methods:**

We analyzed data on product-related injuries in children aged 1 to 17 years from the Xiamen Injury Surveillance System between 2016 and 2024. The study period was categorized into three phases: pre-pandemic, during NPIs, and post-NPIs. Multivariable logistic regression models were constructed, adjusting for demographic and injury-related confounders. Interaction terms (period × injury location, period × product category) were included to analyze the independent effect of the NPIs period on the risk of severe injury (requiring hospital admission) and its effect modification.

**Results:**

A total of 39,245 cases were included. During the NPIs period, the proportion of injuries occurring at home peaked at 56.38%, with notable increases in the proportions of foreign body injuries and burns/scalds. In the post-NPIs period, the proportion of injuries occurring at schools and public places rebounded to 18.07%. While the NPIs period was not independently associated with injury severity in the main effects model, interaction analysis revealed that, compared to furniture-related injuries in the pre-pandemic period, the risk of severe injury was significantly higher during the NPIs period for injuries involving agro-forestry-fishery products (aOR = 15.59, 95% CI: 4.70–51.75), household appliances (aOR = 4.20, 95% CI: 1.37–12.88), and children’s toys (aOR = 3.47, 95% CI: 1.29–9.33). Conversely, the severity risk of injuries occurring at schools and public places in the post-NPIs period was significantly lower than in the pre-pandemic period (aOR = 0.47, 95% CI: 0.23–0.99).

**Conclusion:**

NPIs reshaped the risk landscape of childhood injuries, significantly increasing the severity of injuries associated with specific home-related products (e.g., toys and household appliances). These findings underscore the necessity of integrating targeted product safety interventions within the home environment during public health emergency responses.

## 1 Introduction

Childhood injury is one of the leading causes of death and disability among children globally, constituting a major public health concern (1). Product-related injuries represent a significant component of childhood injury and are closely associated with children’s developmental stages, behavioral characteristics, and environmental exposures (2). The widespread implementation of non-pharmaceutical interventions (NPIs) such as school closures, stay-at-home orders, and restrictions on gatherings during the COVID-19 pandemic altered children’s activity spaces and patterns of product contact in a short period, which created a unique natural experiment to investigate the impact of macro-level social policies on the risk of childhood injury. Multiple studies have reported an overall decline in trauma admissions during the NPIs period (3, 4), along with a noticeable shift in the injury spectrum: road traffic injuries and sports-related trauma decreased substantially (5), while unintentional home injuries and violence-related injuries—particularly firearm injuries—increased (6, 7). However, existing research has largely been confined to describing changes in injury rates or proportional distributions, with limited in-depth assessment of changes in injury severity. More importantly, few studies have employed multivariable models to control for demographic and other confounding factors to examine whether the NPIs period independently influenced the risk of injury associated with specific products or locations, and whether effect modification exists. Therefore, utilizing long-term systematic injury surveillance data from Xiamen City, China, this study aims to investigate: (1) the structural changes in the epidemiology of product-related injuries among children—including but not limited to shifts in product types, injury locations, and population distributions—before, during, and after the implementation of NPIs; and (2) whether the NPIs period independently increased the risk of severe injury and modified the associations between product type, injury characteristics, and severe outcomes, after adjusting for demographic and injury-related confounders.

## 2 Materials and methods

### 2.1 Study design and population

This study was a retrospective observational study using data from the Xiamen Injury Surveillance System. Xiamen is a national sentinel site for product injury surveillance in China; its local system covers seven sentinel hospitals, including both general and specialized institutions. We extracted all product-related injury cases involving children aged 1–17 years that occurred between January 1, 2016, and December 31, 2024.

Inclusion criteria were specifically limited to injuries where the causative factor was identified as a “product” according to the classification standards of the China National Product Injury Information Monitoring System. For the purposes of this study, a “product” is defined as any manufactured item produced to fulfill specific consumer needs. To ensure data independence, duplicate entries or follow-up visits for the same injury event were excluded.

The Institutional Review Board of the Xiamen Center for Disease Control and Prevention exempted this retrospective analysis of de-identified surveillance data from formal ethics approval and individual informed consent.

### 2.2 Variable definitions and classification

Exposure Variable (Period Definition): Based on the timeline of China’s COVID-19 prevention and control policies, the study period was divided into three phases: Pre-NPIs period (2016–2019); During-NPIs period (2020–2022, when NPIs were in effect); Post-NPIs period (2023–2024, after the lifting of restrictive measures).

Outcome Variable: Injury Severity. The primary outcome was a binary measure of injury severity. In alignment with national public health surveillance guidelines in China (8) and international indicators for serious injury (9), we defined a “severe injury” as any case that required inpatient-level healthcare resources. Specifically, this included cases with a final disposition of hospitalization, extended observation in the emergency department, transfer to a higher-level facility, or death. Cases that were treated and discharged directly were classified as “non-severe.”

Covariates: Based on Erikson’s psychosocial development theory and the epidemiological characteristics of childhood injuries, participants were categorized into four developmental stages: toddlerhood (1–3 years), preschool age (4–6 years), school age (7–12 years), and adolescence (13–17 years). Other Covariates included sex, cause of injury, location of injury, and major product category involved (coded according to the classification standards of the General Administration of Quality Supervision, Inspection and Quarantine) (10).

### 2.3 Quality control

All sentinel hospitals use the standardized National Injury Surveillance Report Card embedded in the medical information system for electronic reporting. To ensure data quality, multi-level quality control measures have been implemented, including annual physician training and assessment, real-time system logic verification, quarterly special checks for missed/incorrect reports, and a semi-annual on-site supervision and feedback mechanism.

### 2.4 Statistical analysis

Categorical variables are presented as frequencies (*n*) and percentages (%), group comparisons were conducted using Pearson’s chi-square (*χ*^2^) tests. Variable selection for the multivariable model was based on a combination of statistical and conceptual considerations. First, univariable logistic regression was performed for each candidate variable (sex, age group, injury cause, location, product category, and pandemic period). Although a *p*-value threshold of < 0.2 is sometimes used for preliminary screening, we adopted a full-model approach that included all pre-specified variables of primary interest. This ensured that the estimates for key variables (particularly sex and the NPIs period, which are central to our research questions) were adjusted for all other covariates, regardless of their univariable statistical significance. Subsequently, to evaluate independent associations while controlling for potential confounders, we constructed a multivariable logistic regression model including all the above variables (Multivariable Main Effects Model, hereafter referred to as Model 1). Multicollinearity among independent variables was assessed using Variance Inflation Factors (VIF) before conducting the multivariable logistic regression. A VIF value < 10 was generally considered acceptable, while higher values associated with high-prevalence dummy variables were further evaluated by examining the stability of their standard errors and confidence intervals. To test our primary hypothesis regarding effect modification, we then built upon Model 1 by incorporating three pre-specified interaction terms: (1) age group × period, (2) injury location × period, and (3) major product category × period. This formed the Multivariable Interaction Model (hereafter Model 2), which aimed to examine whether the NPIs period modified the associations of age, injury location, and product category with injury severity.

Model goodness-of-fit was assessed using the Likelihood Ratio Test (LRT) and the Hosmer–Lemeshow test. To mitigate bias in parameter estimation due to sparse data, injury causes and locations with extremely low sample sizes were logically merged prior to analysis (“firearm injury, other, unspecified” were combined into “other/unspecified”; “industrial/construction sites, other, unspecified” were combined into “other/unspecified”).

To evaluate the robustness of our findings regarding the impact of NPIs on injury severity, we conducted two sensitivity analyses: (1) Considering the unique injury patterns in infants, we expanded the age range to include children under 1 year old and refitted the interaction model to examine whether the observed risk patterns were consistent across the entire pediatric age spectrum. (2) To minimize potential clinical heterogeneity in the definition of “severe injury,” we adopted a stricter clinical outcome definition, limiting “severe injury” to only “hospitalization, transfer, or death” cases (*n* = 571), excluding cases requiring only extended observation. Given the limited number of events under this stricter definition, which could lead to overfitting in a multivariable logistic regression model with numerous covariates, this analysis primarily utilized univariate methods. We calculated crude injury proportions across groups and derived crude odds ratios (cORs) using cross-tabulation.

Statistical analyses were performed using SPSS version 25.0 (International Business Machines Corporation) and R version 4.5.1 (R Foundation for Statistical Computing). All statistical tests were two-sided, with a significance level (*α*) set at 0.05.

## 3 Results

### 3.1 Basic information

From 2016 to 2024, a total of 39,245 product-related injury cases among children aged 1 to 17 years were reported by the sentinel hospitals of the injury surveillance system in Xiamen City. Among these, there were 25,157 male cases (64.10%) and 14,088 female cases (35.90%), with a sex ratio of 1.97:1. The top five major product categories involved were: furniture (14,127 cases, 36.00%), stationery, educational and sports supplies (5,843 cases, 14.89%), transportation equipment other than automobiles (4,626 cases, 11.97%), children’s toys and related items (3,317 cases, 8.45%), and hardware and building materials (2,700 cases, 6.88%), collectively accounting for 78.00% (30,613 cases) of all product-related injury cases. Stratified by the pandemic period, there were 20,933 cases (53.34%) in the pre-NPIs (baseline) period, 8,817 cases (22.47%) during the NPIs period, and 9,495 cases (24.19%) in the post-NPIs period.

### 3.2 Distribution of injury characteristics across periods

Across the three periods, the distributions of all characteristics differed significantly (all *p* < 0.001) except for sex, As periods progressed, the proportion of young children (1–3 years) decreased, whereas older children (7–17 years) accounted for a growing share of injuries. During the NPIs period, injuries were increasingly concentrated at home (peak at 56.38%) and involved children’s toys and household daily necessities more frequently, while injuries related to transportation and occurring at schools/public places reached their lowest proportions. The injury cause spectrum shifted accordingly, with burns/scalds and foreign body injuries becoming more prominent, and transport-related causes declining (Table 1).

**Table 1:** Distribution of demographic and injury characteristics of children with product-related injuries across different NPIs periods.

Variable	Pre-NPIs (*N* = 20,933)	During-NPIs (*N* = 8,817)	Post-NPIs (*N* = 9,495)	Total (*N* = 39,245)	*χ* ^2^	*P*
Sex						
Male	13,398 (64.00)	5,639 (63.96)	6,120 (64.45)	25,157 (64.10)	0.68	0.711
Female	7,535 (36.00)	3,178 (36.04)	3,375 (35.55)	14,088 (35.90)		
Age						
1–3 years	8,479 (40.51)	3,183 (36.10)	2,267 (23.88)	13,929 (35.49)	**1,195.50**	<0.001
4–6 years	5,371 (25.66)	2,283 (25.89)	2,174 (22.90)	9,828 (25.04)		
7–12 years	4,857 (23.20)	2,276 (25.81)	3,240 (34.12)	10,373 (26.43)		
13–17 years	2,226 (10.63)	1,075 (12.19)	1,814 (19.10)	5,115 (13.03)		
Injury causes						
Motor vehicle crash	1,280 (6.11)	368 (4.17)	562 (5.92)	2,210 (5.63)	**713.42**	<0.001
Non-motor vehicle crash	977 (4.67)	389 (4.41)	731 (7.70)	2,097 (5.34)		
Fall	7,496 (35.81)	3,021 (34.26)	3,030 (31.91)	13,547 (34.52)		
Blunt injury	7,732 (36.94)	2,985 (33.86)	3,253 (34.26)	13,970 (35.60)		
Cut/Sharp object injury	1,945 (9.29)	1,025 (11.63)	1,236 (13.02)	4,206 (10.72)		
Firearm injury	18 (0.09)	9 (0.10)	6 (0.06)	33 (0.08)		
Burn/scald	314 (1.50)	175 (1.98)	123 (1.30)	612 (1.56)		
Foreign body injury	821 (3.92)	690 (7.83)	310 (3.26)	1,821 (4.64)		
Poisoning	171 (0.82)	74 (0.84)	37 (0.39)	282 (0.72)		
Other	97 (1.34)	45 (1.37)	122 (1.67)	264 (1.43)		
Unspecified	82 (0.39)	36 (0.41)	85 (0.90)	203 (0.52)		
Injury locations						
Home	11,396 (54.44)	4,971 (56.38)	4,159 (43.80)	20,526 (52.30)	**1,180.93**	<0.001
Road/Street	3,063 (14.63)	999 (11.33)	1,538 (16.20)	5,600 (14.27)		
Public residence	2,935 (14.02)	1,429 (16.21)	1,035 (10.90)	5,399 (13.76)		
School and public places	1,989 (9.50)	707 (8.02)	1,716 (18.07)	4,412 (11.24)		
Sports and athletic facility	977 (4.67)	396 (4.49)	554 (5.83)	1,927 (4.91)		
Commercial and service premises	222 (1.06)	70 (0.79)	161 (1.70)	453 (1.15)		
Industrial and construction sites	159 (0.76)	44 (0.50)	28 (0.29)	231 (0.59)		
Other	3 (0.01)	7 (0.08)	15 (0.16)	25 (0.06)		
Unspecified	189 (0.90)	194 (2.20)	289 (3.04)	672 (1.71)		
Injury outcomes						
Treated and discharged	20,441 (97.65)	8,601 (97.55)	9,278 (97.71)	38,320 (97.64)	**67.64**	<0.001
Observation	244 (1.17)	40 (0.45)	70 (0.74)	354 (0.90)		
Hospitalization	208 (0.99)	150 (1.70)	124 (1.31)	482 (1.23)		
Transfer or death	40 (0.19)	26 (0.29)	23 (0.24)	89 (0.23)		
Product categories						
Furniture	8,094 (38.67)	3,007 (34.10)	3,026 (31.87)	14,127 (36.00)	**748.78**	<0.001
Stationery, educational and sports supplies	2,760 (13.18)	1,249 (14.17)	1,834 (19.32)	5,843 (14.89)		
Transport equipment (excl. automobiles)	2,385 (11.39)	890 (10.09)	1,351 (14.23)	4,626 (11.79)		
Children’s toys and related items	1,616 (7.72)	1,038 (11.77)	663 (6.98)	3,317 (8.45)		
Hardware and building materials	1,275 (6.09)	674 (7.64)	751 (7.91)	2,700 (6.88)		
Household daily necessities	1,317 (6.29)	714 (8.10)	636 (6.70)	2,667 (6.80)		
Automobiles	1,018 (4.86)	328 (3.72)	414 (4.36)	1,760 (4.48)		
Agro-forestry-fishery products	962 (4.60)	247 (2.80)	216 (2.27)	1,425 (3.63)		
Food, drugs and related products	436 (2.08)	197 (2.23)	165 (1.74)	798 (2.03)		
General/special equipment	330 (1.58)	116 (1.32)	117 (1.23)	563 (1.43)		
Textiles, garments and accessories	229 (1.09)	152 (1.72)	149 (1.57)	530 (1.35)		
Household appliances	197 (0.94)	98 (1.11)	74 (0.78)	369 (0.94)		
Other products	314 (1.50)	107 (1.21)	99 (1.04)	520 (1.33)		

Data are presented as frequency (percentage) [*n* (%)]. Group comparisons were performed using Pearson’s chi-square test. Bold *χ*^2^ values indicate statistical significance (*p* < 0.05). NPIs, Non-Pharmaceutical Interventions.

### 3.3 Analysis of factors influencing injury severity

The crude associations (cORs) from the univariable analysis are presented alongside the adjusted results in Table 2.

**Table 2:** Factors associated with severe product-related injuries (requiring hospital admission) in children: univariable and multivariable analyses.

Variable	Classification	Univariate analysis			Multivariate analysis		
		cOR	95% CI	*P*	aOR	95% CI	*P*
Sex	Male	1.00			1.00		
	Female	0.98	0.86–1.12	0.779	**0.82**	0.71–0.96	0.011
Age groups	1–3 years old	1.00			1.00		
	4–6 years old	0.86	0.72–1.01	0.065	1.03	0.86–1.24	0.743
	7–12 years old	0.62	0.52–0.75	<0.001	**0.72**	0.58–0.88	0.002
	13–17 years old	1.02	0.84–1.24	0.872	0.89	0.71–1.12	0.320
Cause of injury	Motor vehicle accident	1.00			1.00		
	Fall	0.30	0.24–0.37	<0.001	**0.57**	0.37–0.87	0.010
	Cut/sharp object injury	0.09	0.06–0.14	<0.001	**0.14**	0.07–0.26	<0.001
	Non-motor vehicle crash	0.41	0.29–0.56	<0.001	**0.46**	0.32–0.66	<0.001
	Blunt injury	0.09	0.07–0.12	<0.001	**0.18**	0.11–0.29	<0.001
	Burn/scald	0.22	0.11–0.44	<0.001	**0.22**	0.09–0.50	<0.001
	Poisoning	21.96	16.33–29.52	<0.001	**34.58**	18.99–62.97	<0.001
	Foreign body injury	1.91	1.51–2.41	<0.001	**4.65**	2.80–7.75	<0.001
	Other/unspecified	1.49	1.04–2.14	0.031	**2.27**	1.32–3.89	0.003
Location of the injury	Home	1.00			1.00		
	Road/street	1.59	1.347–1.88	<0.001	1.07	0.69–1.68	0.757
	Public residence	0.43	0.32–0.56	<0.001	0.74	0.54–1.02	0.062
	Schools and public places	0.49	0.37–0.66	<0.001	0.97	0.70–1.35	0.868
	Sports and athletic facility	0.99	0.73–1.35	0.950	**1.93**	1.28–2.90	0.002
	Commercial and service premises	1.62	0.99–2.64	0.057	**2.23**	1.30–3.84	0.004
	Other/unspecified	3.12	2.39–4.08	<0.001	1.13	0.81–1.56	0.482
Period	Pre-NPIs	1.00			1.00		
	During-NPIs	1.04	0.89–1.23	0.607	0.96	0.80–1.15	0.630
	Post-NPIs	0.97	0.83–1.14	0.728	1.13	0.95–1.35	0.180
Product category	Furniture	1.00			1.00		
	Stationery, educational and sports supplies	1.57	1.20–2.05	0.001	1.31	0.94–1.82	0.112
	Transport equipment (excl. automobiles)	3.05	2.41–3.87	<0.001	**2.14**	1.46–3.13	<0.001
	Children’s toys and related items	1.27	0.90–1.80	0.174	**0.36**	0.23–0.55	<0.001
	Hardware and building materials	0.89	0.58–1.37	0.598	1.65	1.001–2.72	0.05
	Household daily necessities	5.29	4.16–6.72	<0.001	**2.03**	1.44–2.84	<0.001
	Automobiles	5.35	4.08–7.00	<0.001	**2.75**	1.76–4.28	<0.001
	Agro-forestry-fishery products	1.48	0.94–2.35	0.094	**0.57**	0.33–0.97	0.037
	Food, drugs and related products	14.86	11.41–19.37	<0.001	1.31	0.82–2.09	0.260
	General/special equipment	3.84	2.41–6.13	<0.001	**3.98**	2.39–6.62	<0.001
	Textiles, garments and accessories	3.89	2.42–6.27	<0.001	**2.48**	1.46–4.21	0.001
	Household appliances	9.74	6.57–14.45	<0.001	**6.32**	3.92–10.19	<0.001
	Other products	14.17	10.41–19.29	<0.001	**3.23**	1.95–5.35	<0.001

cOR, Crude Odds Ratio from univariable analysis. aOR, Adjusted Odds Ratio (adjusted for sex, age group, injury cause, injury location, major product category, and pandemic period) from the Multivariable Main Effects Model (Model 1). All odds ratios are presented with the first category of each variable as the reference. Adjusted odds ratios (aORs) in bold indicate statistical significance (*P* < 0.05). CI, confidence interval; NPIs, Non-Pharmaceutical Interventions.

#### 3.3.1 Main effects model (model 1)

The overall multivariate logistic regression model demonstrated significance (Omnibus test *p* < 0.001) and an adequate fit (Hosmer–Lemeshow test *p* = 0.718). Collinearity diagnostics revealed that most VIF values were < 5, except for ‘falls’ (VIF = 11.8) and ‘blunt injuries’ (VIF = 12.2). These elevated VIFs were deemed structural due to the high frequency of these injury causes (collectively >70%). Model stability was confirmed by the narrow 95% confidence intervals for these estimates (e.g., 0.37–0.87 for falls), indicating no substantial impact on the reliability of the results. Notably, the NPIs period itself was not significantly associated with injury severity in this main effects model. However, several other factors, namely sex, age, injury cause, injury location and product category, were identified as independent predictors (Table 2).

Regarding demographics, females and children aged 7–12 years (vs. 1–3 years) had a lower risk of severe injury.

Injury location was a significant predictor, independent of the NPIs period. Compared with injuries at home, those occurring at commercial/service premises and sports/athletic facilities were associated with approximately two-fold higher odds of risks.

The risk profile varied markedly across product categories, even after adjusting for the pandemic period. Injuries involving household appliances posed the highest risk. Other categories associated with increased severity included general/special equipment, automobiles, and textiles/garments/accessories. In contrast, children’s toys and agro-forestry-fishery products were associated with a reduced risk.

In line with the adjusted odds ratios presented in Table 2, falls and blunt trauma were associated with a lower risk of severe injury compared to motor vehicle accidents. Complete adjusted odds ratios for all variables, including other injury causes, are presented in Table 2.

#### 3.3.2 Effect modification analysis (model 2)

**Figure 1:**
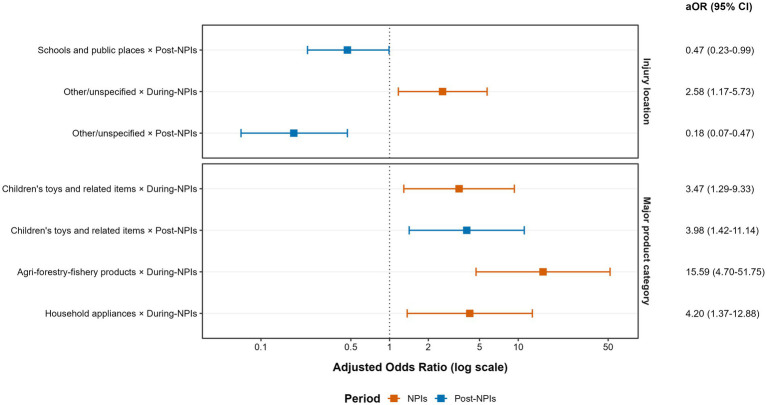
Effect modification by the NPIs period on the association of injury location and product category with the risk of severe injury in children. The figure presents adjusted odds ratios (aORs) and 95% confidence intervals (error bars) for key interaction terms (“injury location × period” and “major product category × period”) from the multivariable logistic regression interaction model (Model 2). All aORs were adjusted for sex, age group, cause of injury, injury location (in product interaction analysis), major product category (in location interaction analysis), and pandemic period (as a main effect). The reference group is set as: injury location = “Home,” product category = “Furniture,” period = “Pre-NPIs.” NPIs, Non-Pharmaceutical Interventions; aOR, adjusted odds ratio; CI, confidence interval.

The Likelihood Ratio Test indicated that Model 2 provided a significantly better fit than Model 1 (*χ*^2^ = 130.74, df = 42, *p* < 0.001). Specifically, the interactions between period and injury location and between period and product category were statistically significant (both *P* for interaction < 0.001), indicating that the NPIs period modified the associations of these factors with injury severity. In contrast, the interaction between age group and period was not significant (*p* = 0.074), suggesting that the effect of age on injury severity did not vary meaningfully across the three periods. Model estimates showed that compared to the reference group (home × pre-NPIs), injuries occurring at schools and public places during the post-NPIs period were associated with a significant 53% reduction in the risk of severity (aOR = 0.47, 95% CI: 0.23–0.99). Conversely, during the NPIs period, injuries occurring at “other/unspecified” locations carried a 2.58 times higher risk of hospitalization compared to the pre-NPIs period (95% CI: 1.17–5.73). Furthermore, compared to the reference group (furniture × pre-NPIs), the risk of severe injury was significantly elevated during the NPIs period for injuries involving agro-forestry-fishery products (aOR = 15.59, 95% CI: 4.70–51.75), household appliances (aOR = 4.20, 95% CI: 1.37–12.88), and children’s toys and related items (aOR = 3.47, 95% CI: 1.29–9.33). In the post-NPIs period, the elevated risk associated with children’s toys and related items remained (aOR = 3.98, 95% CI: 1.42–11.14) (Figure 1). The complete set of estimated aORs for all interaction term combinations derived from Model 2 is provided in Supplementary Table 1.

#### 3.3.3 Sensitivity analysis

The results of the sensitivity analyses consistently supported the core findings of the main effects model.

In Sensitivity Analysis 1 (including infants under 1 year old), the interaction effects remained highly stable. For instance, the increased risk associated with “children’s toys and related items” during the NPIs period remained significant (aOR = 3.04, 95% CI: 1.22–7.57), and the protective association for injuries at “schools and public places” in the post-NPIs period persisted (aOR = 0.47, 95% CI: 0.22–0.98).

Sensitivity Analysis 2 (using the stricter outcome definition) demonstrated that the core risk trends revealed in the main analysis remained robust. Firstly, the “strict severe injury rate” was higher during both the NPIs period (2.00%) and the post-NPIs period (1.55%) compared to the pre-NPIs period (1.18%). Regarding key interaction terms, significantly elevated risks for strict severe injury associated with “children’s toys and related items” were observed during both the NPIs and post-NPIs periods compared to the pre-NPIs period, with cOR values of 12.63 (95% CI: 2.90–55.06) and 11.11 (95% CI: 2.39–51.54), respectively. This direction of effect was entirely consistent with the adjusted odds ratios (aOR) in the main analysis. For the “post-NPIs period × schools and public places” group, univariate analysis yielded a cOR of 1.60 (*p* = 0.31), which did not reach statistical significance (Table 3).

**Table 3:** Sensitivity analysis: comparison of key interaction effects under different definitions of severe injury and age inclusion criteria.

Analysis	Risk combination	OR (95% CI)	*P*	Corresponding aOR from model 2 (95% CI)
Sensitivity analysis 1	During-NPIs × Children’s toys and related items	3.04 (1.22–7.57)	0.017	3.47 (1.29–9.33)
	Post-NPIs × Children’s toys and related items	3.32 (1.28–8.61)	0.014	3.98 (1.42–11.14)
	Post-NPIs × School and public places	0.47 (0.22–0.98)	0.045	0.47 (0.23–0.99)
Sensitivity analysis 2	During-NPIs × Children’s toys and related items	12.63 (2.90–55.06)	<0.001	3.47 (1.29–9.33)
	Post-NPIs × Children’s toys and related items	11.11 (2.39–51.54)	<0.001	3.98 (1.42–11.14)
	Post-NPIs × School and public places	1.60 (0.64–3.98)	0.310	0.47 (0.23–1.00)

Sensitivity analysis 1: Presents the adjusted odds ratios (aORs) from the interaction model after extending the age range to include infants (<1 year). aORs were adjusted for sex, age group, injury cause, injury location, product category, and period. The reference groups are consistent with the main analysis. Sensitivity analysis 2: Presents results using a stricter definition of “severe injury” (including only hospitalization, transfer, or death cases). Due to the limited number of outcome events, crude odds ratios (cORs) from univariate analysis (without adjustment for confounders) are reported to avoid model overfitting. The column “Corresponding aOR in Main Analysis” provides results from Model 2 (the interaction model) in the main text for direct comparison. aOR, adjusted odds ratio; cOR, crude odds ratio; CI, confidence interval.

## 4 Discussion

This study utilized large-scale injury surveillance data to elucidate the dynamic impact of COVID-19 NPIs on product-related injuries among children. Our findings reveal that NPIs not only altered the epidemiological distribution of injuries but also reshaped the risk landscape of injury severity, with the NPIs period acting as a powerful effect modifier. This shifting injurious potential suggests that macro-level social policies can fundamentally redistribute health risks across different environments and products.

A key finding is that while the NPIs period showed no significant independent association with injury severity in the main effects model, its interactions with injury location and product category were highly significant. This suggests that NPIs did not exert a uniform “blanket effect” on severity; instead, the lack of a significant main effect likely reflects competing countervailing trends: a substantial reduction in high-risk outdoor injuries (e.g., motor vehicle crashes) due to restricted mobility was statistically offset by a concurrent intensification of injury severity within the home environment.

The restructuring of activity spaces led to a marked concentration of injuries at home (peaking at 56.38%), aligning with the global “lockdown effect” (11, 12). However, our interaction analysis further delineates that the severity risk for injuries involving children’s toys (aOR = 3.47) and household appliances (aOR = 4.20) surged during NPIs. This intensification likely stems from dual mechanisms: “increased exposure dose” due to prolonged confinement and “caregiver fatigue” during remote work, which created supervision gaps and may have delayed intervention for minor incidents (13–17). Unlike pre-pandemic patterns, the home environment under NPI conditions became a site where domestic hazards possessed a higher potential to escalate into severe outcomes.

Conversely, the interaction analysis reveals a significant “Safety Resilience” in public institutions. Despite a post-pandemic rebound in the proportion of school-related injuries (18.07%), which aligns with other research (18), the risk of these injuries resulting in severe outcomes decreased by 53% (aOR = 0.47) compared to the pre-pandemic period. This seemingly paradoxical phenomenon of “high frequency but low severity” suggests that schools and public venues successfully institutionalized strengthened safety protocols and emergency response density during the pandemic (19). In contrast, NPIs triggered a “spillover effect” into informal spaces; when conventional recreational venues were restricted, children shifted toward “other/unclear” locations (e.g., agricultural sites, woodlands) where the severity risk rose significantly (aOR = 2.58) due to a lack of safety regulation and environmental adaptation (20).

Beyond the reshaped risk within built environments, the interaction analysis also captured the severe consequences of population displacement into natural or productive landscapes. The 15-fold surge in severity risk for agro-forestry-fishery products (aOR = 15.59) during NPIs highlights the impact of large-scale decentralized migration. Multiple international studies have confirmed a significant “decentralized” migration trend during COVID-19 lockdowns, with a massive outflow of people from high-density urban centers to suburban or rural areas (21, 22). That outflow exposed children to unfamiliar farming tools and more hazardous natural environments (23, 24). This “environmental mismatch” underscores that population shifts under restrictive measures require targeted safety education to bridge the gap between children’s prior experience and their new, more hazardous surroundings.

The robustness of these conclusions is supported by our multi-dimensional sensitivity analyses. The consistency of findings under a stricter outcome definition (Sensitivity Analysis 2) serves as a rigorous internal validation, confirming that the observed effect modifications are not artifacts of injury classification but reflect substantive changes across different clinical thresholds. Furthermore, the non-significant interaction between age group and period suggests that while NPIs altered activity scope, they did not modify the inherent developmental susceptibility to injury across the pediatric age spectrum.

This study has clear implications for public health practice. Prevention strategies should transition from generalized “home safety” to “precision intervention,” emphasizing adherence to toy safety standards, proactive safeguarding of household appliances, and environmental adaptation for families relocating during emergencies. Moreover, the experience of reduced injury severity in public places post-pandemic warrants institutionalization into sustainable safety standards.

This study also has several limitations. First, data were sourced from a single regional surveillance system, warranting caution regarding generalizability. Second, although we controlled for multiple confounders using multivariable models, unmeasured variables such as quality of home care, parental psychological stress, and socioeconomic status could not be included, potentially leading to residual confounding. Furthermore, as an observational study based on administrative data, we could not directly measure specific behavioral mediators of NPIs (e.g., actual time children spent at home, duration of online learning, or outdoor activity frequency). Therefore, although the observed changes in injury patterns (e.g., increased home injuries, decreased outdoor transport injuries, increased risk from home-related products) are highly consistent with the theoretical mechanism of “activity space shift to home” and align with global trends, alternative explanations cannot be entirely ruled out. This limitation primarily affects the in-depth interpretation of causal pathways but has a relatively limited impact on the main finding of a strong association between the NPIs period and injury patterns. Lastly, defining the entire 2020–2022 period as the “NPIs implementation period,” while based on the overarching presence of various NPIs, did not capture temporal variations in NPI intensity. China’s prevention policies followed a “dynamic zeroing” principle, encompassing forms ranging from comprehensive strict lockdowns to precise zonal control (25). This heterogeneous exposure definition may have led to a degree of non-differential misclassification, which typically biases effect estimates toward the null (i.e., dilution bias), potentially underestimating the true modifying effect of NPIs. However, this definition aligns with our research objective of examining the macro-policy “overall shift”. Future research could integrate multi-regional data and incorporate individual-level behavioral measurements, while adopting the ‘Stringency Index’ from the Oxford COVID-19 Government Response Tracker (OxCGRT) to further elucidate the underlying dynamic causal pathways (26).

## 5 Conclusion

In summary, COVID-19 NPIs dynamically reshaped the risk determinants of product-related injuries in children. By modifying the injurious potential of specific settings and products, NPIs led to a sharp increase in home-related injury severity while potentially catalyzing long-term improvements in public environmental safety. These findings call for the integration of targeted, context-specific injury prevention strategies within future public health emergency responses.

## Data Availability

The data analyzed in this study is subject to the following licenses/restrictions: The data underlying this study are managed by the Xiamen Center for Disease Control and Prevention (Xiamen CDC) as part of a confidential public health surveillance system. To protect patient privacy and comply with data governance regulations, they are not publicly deposited. As stated in our Data Availability Statement, requests for access should be directed to the Xiamen CDC, subject to their review and approval based on a justified research proposal. Requests to access these datasets should be directed to Youlan Chen, 89274900@qq.com.
