# Supplementary material for: Epidemiological characteristics and risk factors for severity of product-related injuries in children during COVID-19: a non-pharmaceutical intervention study
Source: Front Public Health. 2026 Feb 20;14:1783277. doi: 10.3389/fpubh.2026.1783277 (PMC12963239; doi:10.3389/fpubh.2026.1783277)
Supplement: Supplementary file 1 [file Data_Sheet_1.pdf]

Supplementary Table 1

**Variable**	**Classification**	**aOR**	**95%CI**	***P***
Age group × Period	1-3 years × Pre-NPIs	1.00		
	4-6 years × During-NPIs	1.49	0.94-2.37	0.091
	4-6 years × Post-NPIs	1.33	0.83-2.15	0.238
	7-12years × During-NPls	1.61	0.97-2.68	0.065
	7-12 years × Post-NPIs	1.09	0.64-1.83	0.760
	13-17 years × During-NPls	0.67	0.35-1.29	0.232
	13-17 years × Post-NPIs	1.21	0.69-2.11	0.508
Injury location × Period	Home × Pre-NPIs	1.00		
	Road/Street × During-NPIs	0.64	0.24-1.69	0.367
	Road/Street × Post-NPIs	0.79	0.32-1.96	0.603
	Public residence × During-NPIs	0.50	0.21-1.20	0.122
	Public residence × Post-NPIs	0.58	0.26-1.27	0.173
	Schools & public places × During-NPIs	0.82	0.33-2.04	0.669
	Schools & public places × Post-NPIs	**0.47**	0.23-1.00	0.049
	Sports & Athletic facility × During-NPIs	1.15	0.41-3.24	0.792
	Sports & Athletic facility × Post-NPIs	0.39	0.15-1.06	0.064
	Commercial & Service premises × During-NPIs	1.69	0.34-8.30	0.521
	Commercial & Service premises × Post-NPIs	1.35	0.39-4.63	0.635
	Other/unspecified × During-NPIs	**2.58**	1.17-5.73	0.019
	Other/unspecified × Post-NPIs	**0.18**	0.07-0.47	0.000
Product Category × Period	Furniture × Pre-NPIs	1.00		
	Stationery, Educational & Sports supplies × During-NPIs	0.77	0.32-1.88	0.570
	Stationery, Educational & Sports supplies × Post-NPIs	1.32	0.62-2.77	0.471
	Transport equipment (excl. automobiles) × During-NPIs	1.19	0.43-3.28	0.734
	Transport equipment (excl. automobiles) × Post-NPIs	0.78	0.31-1.97	0.594
	Children's toys & Related items × During-NPIs	**3.47**	1.29-9.33	0.014
	Children's toys & Related items × Post-NPIs	**3.98**	1.42-11.14	0.009
	Hardware & building materials × During-NPIs	1.58	0.56-4.47	0.392
	Hardware & building materials × Post-NPIs	0.33	0.09-1.26	0.104
	Household daily necessities × During-NPIs	1.53	0.79-2.97	0.204
	Household daily necessities × Post-NPIs	1.91	0.99-3.69	0.055
	Automobiles × During-NPIs	1.36	0.44-4.19	0.597
	Automobiles × Post-NPIs	0.75	0.27-2.14	0.594
	Agro-forestry-fishery products × During-NPIs	**15.59**	4.70-51.75	0.000
	Agro-forestry-fishery products × Post-NPIs	2.82	0.60-13.32	0.190
	Food, Drugs & Related products × During-NPIs	0.49	0.21-1.15	0.101
	Food, Drugs & Related products × Post-NPIs	1.05	0.46-2.38	0.907
	General/Special equipment × During-NPls	2.55	0.74-8.75	0.138
	General/Special equipment × Post-NPIs	1.40	0.37-5.28	0.620
	Textiles, Garments & Accessories × During-NPIs	1.39	0.42-4.65	0.592
	Textiles, Garments & Accessories × Post-NPIs	0.74	0.21-2.66	0.644
	Household appliances × During-NPls	**4.20**	1.37-12.88	0.012
	Household appliances × Post-NPIs	2.18	0.66-7.22	0.203
	Other products × During-NPIs	0.33	0.10-1.09	0.068
	Other products × Post-NPIs	0.88	0.25-3.10	0.841

Note: Adjusted odds ratios (aORs) in bold indicate statistical significance ($P < 0.05$). All odds ratios are presented with the first category of each variable as the reference.Abbreviations: aOR, adjusted odds ratio; CI, confidence interval; NPIs, Non-Pharmaceutical Interventions.
